# Superconductivity of novel tin hydrides (Sn_*n*_H_*m*_) under pressure

**DOI:** 10.1038/srep22873

**Published:** 2016-03-11

**Authors:** M. Mahdi Davari Esfahani, Zhenhai Wang, Artem R. Oganov, Huafeng Dong, Qiang Zhu, Shengnan Wang, Maksim S. Rakitin, Xiang-Feng Zhou

**Affiliations:** 1 Department of Geosciences, Center for Materials by Design, and Institute for Advanced Computational Science, State University of New York, Stony Brook, NY 11794-2100, USA; 2 Peter Grunberg Research Center, Nanjing University of Posts and Telecommunications, Nanjing, Jiangsu 210003, China; 3 Skolkovo Institute of Science and Technology, Skolkovo Innovation Center, 3 Nobel St., Moscow 143026, Russia; 4 Department of Problems of Physics and Energetics, Moscow Institute of Physics and Technology, 9 Institutskiy Lane, Dolgoprudny City, Moscow Region 141700, Russia; 5 International Center for Materials Discovery, School of Materials Science and Engineering, Northwestern Polytechnical University, Xi’an, Shaanxi 710072, People’s Republic of China; 6 School of Physics and Key Laboratory of Weak-Light Nonlinear Photonics, Nankai University, Tianjin 300071, China

## Abstract

With the motivation of discovering high-temperature superconductors, evolutionary algorithm USPEX is employed to search for all stable compounds in the Sn-H system. In addition to the traditional SnH_4_, new hydrides SnH_8_, SnH_12_ and SnH_14_ are found to be thermodynamically stable at high pressure. Dynamical stability and superconductivity of tin hydrides are systematically investigated. I
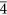
m2-SnH_8_, C2/m-SnH_12_ and C2/m-SnH_14_ exhibit higher superconducting transition temperatures of 81, 93 and 97 K compared to the traditional compound SnH4 with T*c* of 52 K at 200 GPa. An interesting bent H_3_–group in I
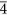
m2-SnH_8_ and novel linear H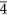 in C2/m-SnH_12_ are observed. All the new tin hydrides remain metallic over their predicted range of stability. The intermediate-frequency wagging and bending vibrations have more contribution to electron-phonon coupling parameter than high-frequency stretching vibrations of H2 and H3.

Molecular hydrogen’s phase transition to a metallic state has been subject of many experimental and theoretical studies^1^,^2^. Although reaching the metallic state in pure solid hydrogen proved elusive, it is in the main focus of many groups and recently, the progress of bringing pure hydrogen to nearly 400 GPa has been reported^3^,^4^,^5^. Following the pioneering work of Ashcroft^6^, nearly room-temperature superconductivity was predicted in metallic molecular hydrogen^7^,^8^.

An alternative approach to metalize hydrogen is to use chemical alloying as a means to exert additional pressure on hydrogen atoms^9^. Hydrogen-rich compounds such as SiH_4_ can be metalized at a much lower pressure^10^. For metallic hydrogen, high Debye temperature and strong electron-phonon coupling are anticipated. The same is expected for hydrogen-rich compounds and it has been suggested that hydrogen-rich compounds are good candidates for high-temperature superconductivity^9^ Theoretical studies confirm this idea with predicting superconductivity in high-pressure hydrides such as H-Se^11^, Ca-H^12^, Sn-H^13^, Pt-H^14^ and B-H^15^. A series of hydrogen-rich compounds have been predicted to have remarkably high T_*c*_ values (e.g. 235 K for CaH_6 _at 150 GPa^12^, 191 K for H_3_S at 200 GPa^16^, 64 K for GeH_4_ at 220 GPa^17^) while the highest T_*c*_ that had been achieved experimentally was in the complex mercury cuprate (138 K at ambient pressure^18^ and 166 K at high pressures^19^). The new record of high T_*c*_ was established for H_3_S, a compound whose existence and superconductivity at 200 K were first predicted theoretically^16^ in 2014 using USPEX and then observed experimentally^20^ in 2015, and started a new wave of interest in hydrogen-rich superconductors.

In a previous theoretical study, Tse *et al.* reported a high-pressure metallic phase of SnH_4_ with hexagonal P6/mmm symmetry group, which is a layered structure intercalated with H_2_ units, and is a superconductor with Tc close to 80 K at 120 GPa^21^. Later, by using evolutionary algorithm USPEX, Gao *et al.*^13^ reported two novel metallic phases of SnH_4_ with space groups P6_3_/mmc and Ama2, which both have hexagonal layers of Sn atoms with semi-molecular H_2_ units. The reported stability ranges are 96–180 GPa for Ama2, and above 180 GPa for P6_3_/mmc; with T_*c*_ values of 15–22 K at 120 GPa and 52–62 K at 200 GPa for Ama2 and P6_3_/mmc, respectively^13^.

While SnH_4_ was shown to be a relatively high-T_*c*_ superconductor, the possibility of existence of other tin hydrides were not explored so far. At the same time, by now it is proven^22^ that totally unexpected compounds become stable under pressure, and this gives hope of finding even better superconductors. Hence, in this study, we systematically search for the stable compounds using the highly efficient variable-composition evolutionary searches (VCES). Apart from the previously reported phases of SnH_4_, there is one metastable tetragonal phase of stannane with higher superconducting critical temperature. Other stable superconducting compounds, SnH_8_, SnH_12_ and SnH_14_, are found to become stable at high pressure. Moreover, we found a semi-molecular group H_3_– in the I
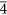
m2 structure of SnH_8_. Novel H_4_–is also present in C2/m-SnH_12_. We calculate a high T_*c*_ of 81 K at 220 GPa in the newly predicted compound SnH_8_, 93 K for SnH_12_ at 250 GPa, 97 K for SnH_14_ at 300 GPa and 91 K for the metastable phase of SnH_4_ at 220 GPa.

## Results

Evolutionary variable-composition searches for stable compounds and their structures with up to 20 atoms in the primitive unit cell were performed at 150, 200, 250 and 300 GPa. To further investigate the newly found compounds, fixed-composition structure predictions for the most promising compounds were performed, with one to three formula units per cell. Candidate low-enthalpy structures are metastable I4/mmm-SnH_4_, stable I
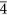
m2-SnH_8_, C2/m-SnH_12_ and C2/m-SnH_14_. In the I
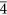
m2-SnH_8_ structure predicted to be stable at pressures above 220 GPa, Sn atoms are packed between H_2_ and H_3_ molecular groups, in which the bent H_3_ units are perpendicular to one another and sepated by 1.35 Å. In C2/m-SnH_12_, Sn atoms form well-separated close-packed layers intercalated with blocks of H_2_ and H_4_ semi-molecules.

**Figure 1:**
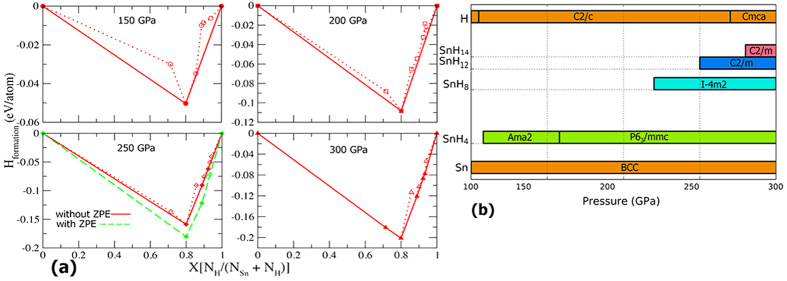
Thermodynamics of the Sn-H system. (**a**) Predicted formation enthalpy of Sn_*n*_H_*m*_ compounds. Solid red lines denote the convex hull and green dashed line shows the effect of ZPE inclusion at 250 GPa. (**b**) Predicted pressure-composition phase diagram of the Sn-H system.

Figure 1(a). shows the enthalpy of formation (Δ*H*) of Sn-H compounds at selected pressures. Significantly, in addition to reproducing various structures of solid SnH_4_^13^,^21^, Sn^23^ and H_2_^24^, novel compounds SnH_8_, SnH_12_ and SnH_14_ are found to be stable in a wide pressure range in our systematic evolutionary structure search. It can be seen from Fig. 1(a). that at around 200 GPa the tetragonal SnH_8_ with the space group of I
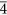
m2 lies above the convex hull, therefore, is metastable with respect to decomposition to P6_3_/mmc-SnH_4_ and C2/c-H_2_. Between 150 to 300 GPa, we predict stable phases of H_2_, SnH_4_, SnH_8_, SnH_12_, SnH_14_ and Sn^23^. Some metastable forms of SnH_6_, SnH_9_ and SnH_16_ are also shown in Fig. 1(a) by open symbols.

SnH_4_ is thermodynamically stable at pressures above 108 GPa as was predicted in previous report^13^. It goes through a phase transition at 160 GPa. Upon increasing pressure, at 220 GPa we predict stabilization of SnH_8_. SnH_12 _and SnH_14_ reach stability at the pressures of 250 GPa and 280 GPa, respectively, and remain stable at least up to 300 GPa. The structures of SnH_*n*_ compounds are found to be dynamically stable within pressure ranges of their stability. In the I
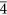
m2-SnH_8_ structure, Sn atoms occupy the 2a Wyckoff site and the H atoms are on the 4e, 8i and 4f sites (detailed structural information is provided in Table 1).

We checked the effects of zero-point energy using phonon calculations^25^ at 250 GPa. The inclusion of zero-point noticeably lowered the formation enthalpy of SnH_8_ with respect to SnH_4_ and H_2_ (Fig. 1(a)), implying that this compound can be formed at lower pressure. At the same time, SnH_12_ moves above the convex hull at 250 GPa, suggesting that higher pressure is needed to stabilize C2/m-SnH_12_. In accord with what we expect, zero-point energy does not change the topology of the phase diagram, but shifts transition pressures.

In I
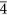
m2-SnH_8_ structure, the H atoms are split into two categories. Some H atoms form H_3_ groups, which were previously observed in solid phases of BaH_6_^27^, in an unstable structure of H_5_Br ([H_3_]Br[H_2_])^28^, and in an intriguing linear form with the bond length of 0.92 Å in H_5_Te_2_^29^. In contrast to H_5_Br, which has approximately an equilateral triangle form of H_3_, here we report the formation of H_3_ in a bent geometry with the angle of 146.2° and bond length of 0.86 Å at 220 GPa in the I
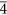
m2 structure. The other type of H atoms form H_2_ groups which are aligned parallel to each other.

**Figure 2:**
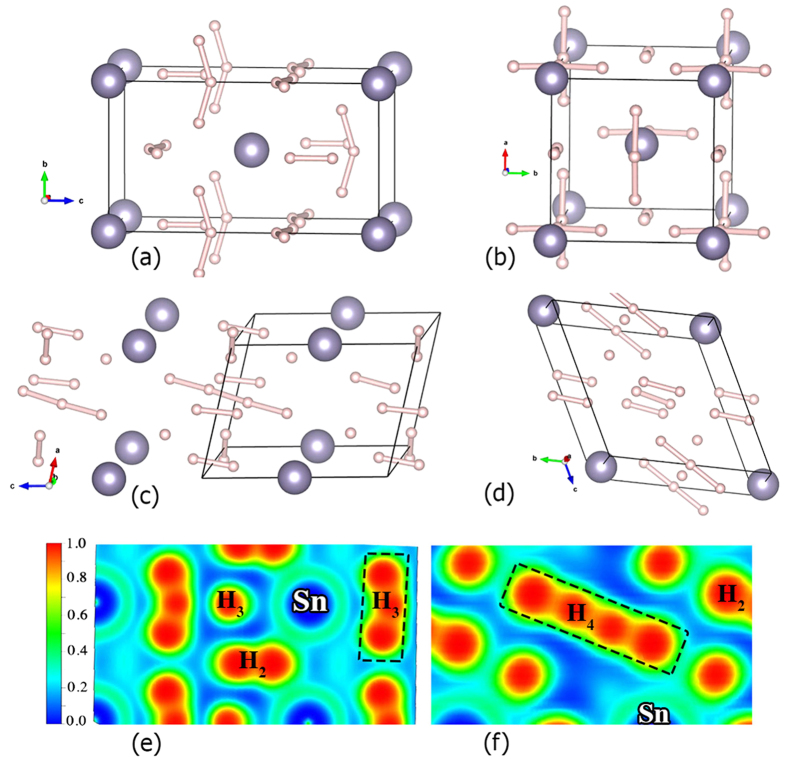
Predicted structures of (**a,b**) SnH_8_ [I
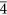
m2], (**c**) SnH_12_ [C2/m] and (**d**) SnH_14_ [C2/m]. Large and small spheres represent Sn and H atoms, respectively. Electron localization functions (ELF) for (**e**) SnH_8_ [I
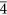
m2] at 220 GPa and (f) SnH_12_ [C2/m] at 250 GPa.

I
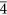
m2 structure can be presented as Sn[H_2_][H_3_]_2_ as shown in Fig. 2(a,b). The bond length in H_3_ unit is 0.86 Å, whereas H_2_ has a longer bond length of 0.87 Å. Contrary to isolated H_2_ molecule, which only has a filled *σ* bonding orbital, in the H_2_ and H_3_ semi-molecules, population of anti-bonding orbitals causes lengthening and weakening of the covalent bond. The slightly longer H-H bond length compared to isolated H_2_ molecule (0.74 Å) is caused by charge transfer of 0.42 e^−^ and 0.48 e^−^, as computed using Bader theory^26^, from Sn to each H_2_ and H_3_ unit, respectively. Charge transfer is an important factor in the formation of H_2_ and H_3_ units in the H_4_Te, GeH_4_, SnH_4_, CaH_4_, H_5_Te_2_, H_5_Br, BaH_6_^12^,^17^,^27^,^28^,^29^

Analysis of electron localization function (ELF) shows a high ELF value of 0.88 between H atoms within the unit, indicating strong covalent bonding features (Fig. 2(e)). At the same time, the ELF value on the Sn-H bond is very low, reaching just 0.37.

In C2/m-SnH_12_, intriguing formation of novel H_4_ semi-molecules are observed; at 250 GPa, they can be represented as two H_2_-groups separated by just 0.99 Å. Higher pressure of 300 GPa decreases the distance to 0.88 Å, leading to a strong covalent bond in the 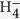 units. Fig. 2(f). demonstrates covalent bonds in the linear H_4_ units with the ELF magnitude of 0.85.

**Figure 3:**
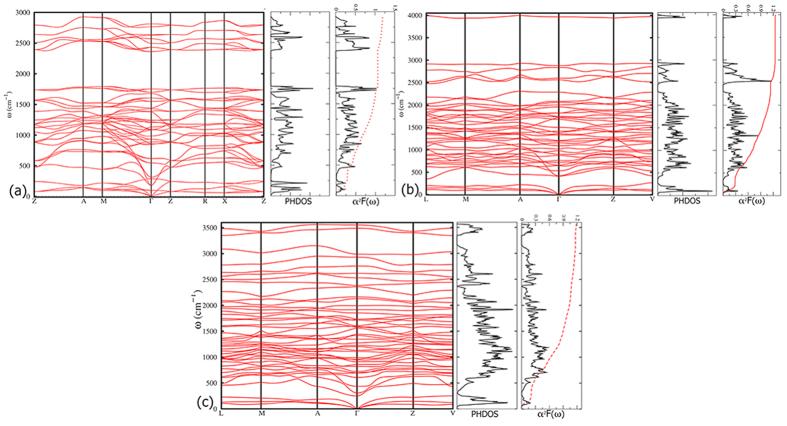
Phonon band structure, phonon DOS, Eliashberg phonon spectral function *α*^2^F(*ω*) and electron-phonon integral *λ*(*ω*) of: (**a**) SnH_8_ [I
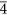
m2] at 220 GPa, (**b**) SnH_12_ [C2/m] at 250 GPa and (**c**) SnH_14_ [C2/m] at 300 GPa.

The calculated phonon dispersion curves and phonon density of states for I
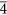
m2 structure of SnH_8_ at 220 GPa are shown in Fig. 3(a). Dynamical stability is clearly evidenced by the absence of any imaginary frequencies in the whole Brillouin zone. The low-frequency bands below 250 cm^−1^ are mainly from the vibrations of Sn atoms. Modes between 300 and 1700 cm^−1^ are mainly from the H-H wagging vibrations, and higher frequency vibrations above 2300 cm^−1^ are due to H-H stretching vibrations in H_2_ and H_3_ units.

Low-frequency translational modes, mostly from Sn atom, contribute 23.7% (9.2%) to the total *λ*. Intermediate H-H wagging vibrations make a significant contribution of 65.7% (67.9%), and the rest is from stretching H vibrations, which contribute 10.6% (22.9%) for SnH_8_ (SnH_12_). This is different from superconductivity in Cmcm-H_2_Br^28^, where Br translational modes make the largest contribution to the total *λ* and similar to the R
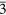
m-H_4_Te^29^ and P4/mmm-BaH_6_^27^, where medium-frequency H-wagging and bending modes contribute the most to the EPC. In accord with our expectation, *λ* increases almost linearly with hydrogen content, where we found 60.2%, 72.2% and 77.1% contribution of H vibrations to the total *λ* of SnH_4_, SnH_8_ and SnH_12_, respectively. This highlights the dominant role of H in the superconductivity of H-rich compounds.

**Figure 4:**
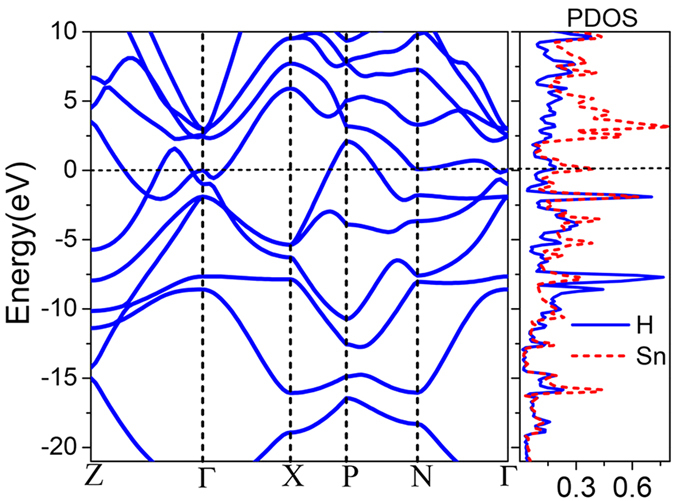
Electronic band structure and projected DOS on Sn and H atoms for SnH_8_ [I
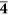
m2] at 220 GPa.

Electronic band structure of I
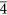
m2-SnH_8_ is depicted in Fig. 4. Occurrence of flat and steep bands near the Fermi level has been suggested as a condition for enhancing electron-phonon coupling (EPC) and the formation of Cooper pairs.

We can calculate T_*c*_ based on the spectral function *α*^2^F(*ω*) and taking advantage of Allen-Dynes modified McMillan equation (Eq. 1.) by using Coulomb pseudo-potential *μ*∗ of 0.10 and 0.13 as widely accepted values (see Table 2). At 220 GPa, the predicted T_*c*_ values for I
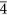
m2-SnH_8_ are 81 K and 72 K using *μ*∗ values of 0.10 and 0.13, respectively. The calculated T_*c*_ slightly decreases with pressure (82 K at 200 GPa and 79 K at 300 GPa using *μ*∗ = 0.10) with a pressure coefficient of −0.023 K/GPa 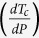. Reported *λ* is comparable to H_3_Se (*λ* = 1.09) at 200 GPa^11^, but in I
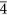
m2-SnH_8_ structure, we have a lower 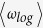 of 919 K (1477 K for H_3_Se), resulting in a lower T_*c*_ value.

In conclusion, we explored the energetically stable/metastable high-pressure phases of the Sn-H system in detail by means of *ab initio* evolutionary structure prediction. The results demonstrate that SnH_8_, SnH_12_ and SnH_14_, reported for the first time in this work, are thermodynamically stable compounds that coexist stably with solid Sn, H_2_ and SnH_4_ in a wide pressure range starting from 220 to at least 300 GPa.

EPC calculations indicate that high-pressure SnH_8_, SnH_12_ and SnH_14_ are phonon-mediated superconductors with T_*c*_ values of 81, 93 and 97 K at pressures of 220, 250, and 300 GPa, respectively. *λ* is high for SnH_*n*_ compounds, comparable with H_3_M-Im
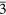
m, where M = S and Se^11^. Structures of SnH_*n*_ compounds contain 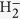, bent 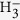, and linear 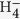 groups. Further experimental studies on the formation of SnH*n*, n = 8, 12 and 14 at high pressure are needed, and present results will serve as a guide for future experiments.

## Methods

To find stable and low-enthalpy metastable structures, we took advantage of evolutionary algorithm implemented in USPEX code^30^,^31^,^32^, which has been extensively used to predict stable crystal structures with just a knowledge of the chemical composition and without any experimental information^15^,^33^,^34^,^35^.

In this method, the initial generation of structures and compositions is produced with the random symmetric algorithm^34^, and subsequent generations are produced by carefully designed variation operators. In order to find all stable stoichiometric compounds and the corresponding stable and metastable structures in the Sn-H binary system, we used VCES method implemented in USPEX^30^,^31^.

Structure relaxations were carried out using VASP package^36^ in the framework of density functional theory (DFT) and using PBE-GGA (Perdew-Burke-Ernzerhof generalized gradient approximation)^37^. The projector-augmented wave approach (PAW)^38^ was used to describe the core electrons and their effects on valence orbitals. The plane-wave kinetic energy cutoff was chosen as 1000 eV for hard PAW potentials, and we used Γ-centered uniform *k*-points meshes to sample the Brillouin zone.

Phonons and thermodynamic properties of Sn-H compounds are calculated using the PHONOPY package^25^,^39^. The supercell approach is used with supercell dimensions greater than 10 Å (typically 3 × 3 × 3 replication of the primitive cell). We used valence electron configurations of 4d^10^ 5s^2^ 5p^2^ and 1s^1^ for tin and hydrogen, respectively. Phonon frequencies and electron-phonon coupling (EPC) coefficients are calculated using DFPT as implemented in the Quantum ESPRESSO (QE) code^40^. In the QE calculations, we employed ultrasoft pseudopotentials and PBE-GGA exchange-correlation functional^37^. A plane-wave basis set with a cutoff of 70 Ry gave a convergence in energy with a precision of 1 meV/atom. The EPC parameter was calculated using 4 × 4 × 3, 5 × 5 × 4 and 5 × 5 × 4 *q*-point meshes for I
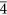
m2-SnH_8_, C2/m-SnH_12_ and C2/m-SnH_14_, respectively. Denser *k*-point meshes, 8 × 8 × 6, 10 × 10 × 8 and 10 × 10 × 8 were used for convergence checks for the EPC parameter *λ*. The superconducting T_*c*_, was estimated using the Allen-Dynes modified McMillan equation^41^:


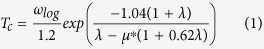


where *ω*_*log*_ is the logarithmic average frequency and *μ*∗ is the Coulomb pseudopotential, for which we used 0.10 and 0.13 values, which often give realistic results. The EPC constant and *ω*_*log*_ were calculated as:


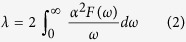


and


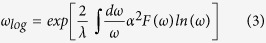


## Additional Information

**How to cite this article**: Mahdi Davari Esfahani, M. *et al.* Superconductivity of novel tin hydrides (Sn_*n*_H_*m*_)under pressure. *Sci. Rep.* **6**, 22873; doi: 10.1038/srep22873 (2016).

## Figures and Tables

**Table 1:** Predicted crystal structures of SnH_8_, SnH_12_ and SnH_14_ at 220, 250 and 300 GPa, respectively.

Phase	Lattice parameters	Atom	x	y	z
I 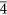 m2	a = 3.076 Å	Sn(2a)	0.0000	0.0000	0.0000
SnH_8_	c = 5.523 Å	H_1_(8i)	0.2729	0.0000	0.3331
	at 220 GPa	H_2_(4e)	0.0000	0.0000	0.6208
		H_3_(4f)	0.0000	0.5000	0.1701
C2/m	a = 5.191 Å	Sn(2d)	0.0000	0.5000	0.5000
SnH_12_	b = 3.065 Å	H_1_(4i)	0.0495	0.0000	0.6553
	c = 7.388 Å	H_2_(4i)	0.4564	0.0000	0.7226
	*β* = 148.95°	H_3_(4i)	0.3428	0.0000	0.8832
	at 250 GPa	H_4_(8i)	0.3810	0.2399	0.1123
		H_5_(4g)	0.0000	0.1233	0.0000
C2/m	a = 7.129 Å	Sn(2b)	0.0000	0.5000	0.0000
SnH_14_	b = 2.730 Å	H_1_(4i)	0.3651	0.0000	0.7031
	c = 3.673 Å	H_2_(4i)	0.1857	0.0000	0.9852
	*β* = 60.71°	H_3_(4i)	0.0732	0.0000	0.6252
		H_4_(4i)	0.8063	0.0000	0.8090
	at 300 GPa	H_5_(8i)	0.2365	0.2808	0.4035
		H_6_(2d)	0.0000	0.5000	0.5000
		H_7_(2c)	0.0000	0.0000	0.5000

**Table 2:** The calculated EPC parameter (*λ*), logarithmic average phonon frequency (*ω*_*log*_) and critical temperature (T_*c*_) (with μ*  =  0.10 and 0.13) for metastable SnH_4_, stable SnH_8_, SnH_12_ and SnH_14_ at 220, 220, 250 and 300 GPa, respectively.

Structure	Pressure (GPa)	*λ*	*ω*_*log*_ (K)	T_*c*_ (K)
I4/mmmSnH_4_	220	1.180	1025	91 (μ* = 0.10)
				80 (μ* = 0.13)
I 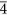 m2-SnH_8_	220	1.188	919	81 (μ* = 0.10)
				72 (μ* = 0.13)
C2/m-SnH_12_	250	1.250	991	93 (μ** *= 0.10)
				83 (μ* = 0.13)
C2/m-SnH_14_	300	1.187	1099	97 (μ** *= 0.10)
				86 (μ** *= 0.13)
